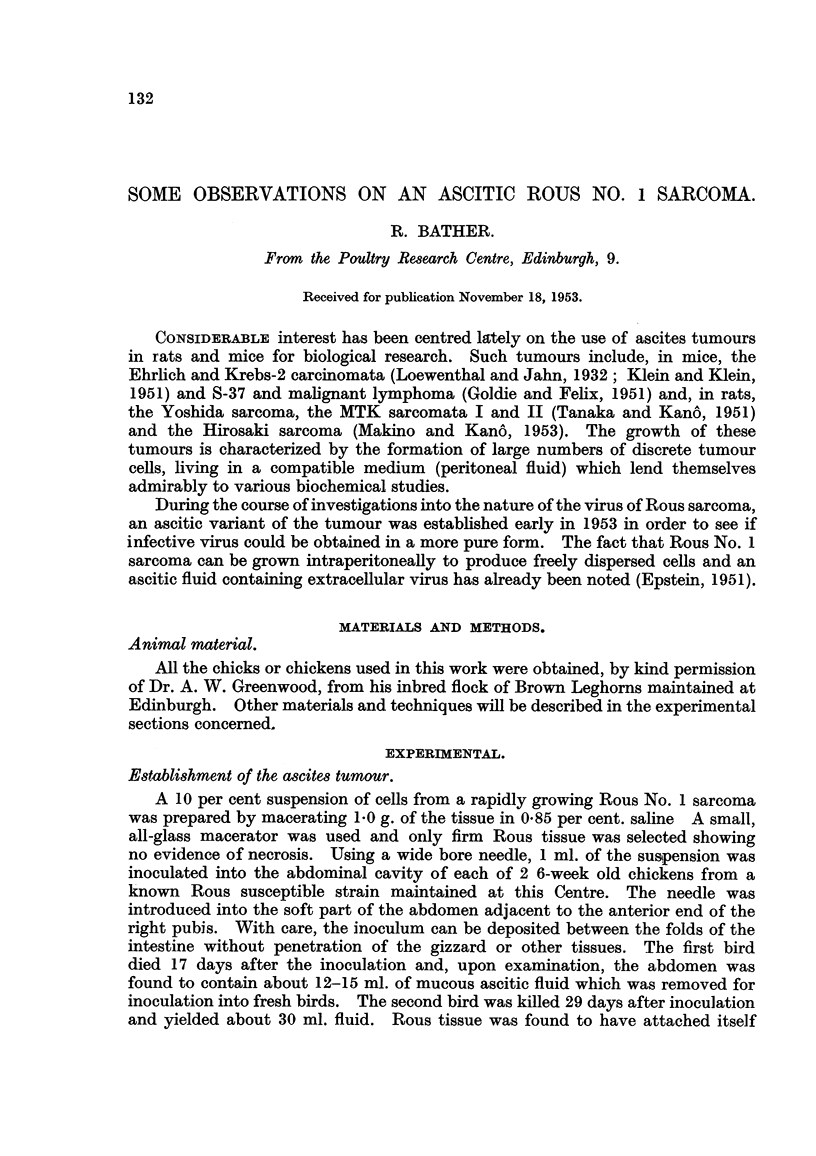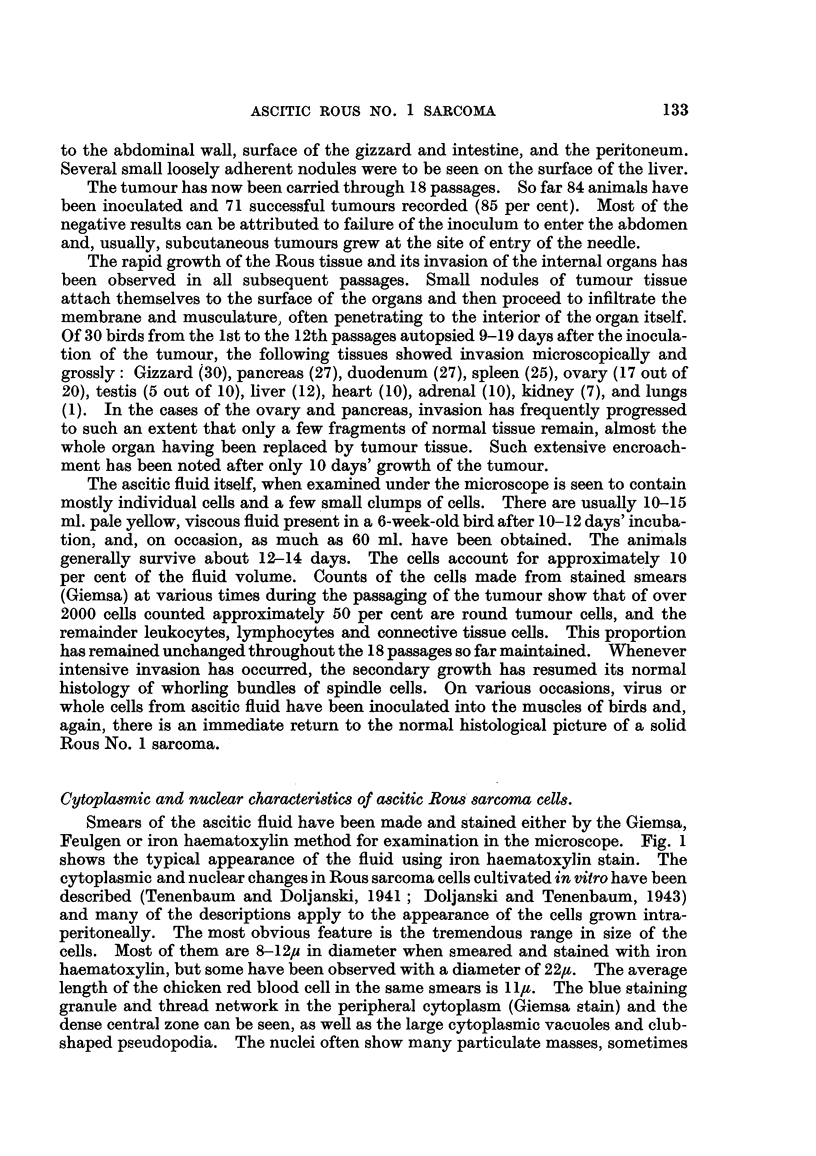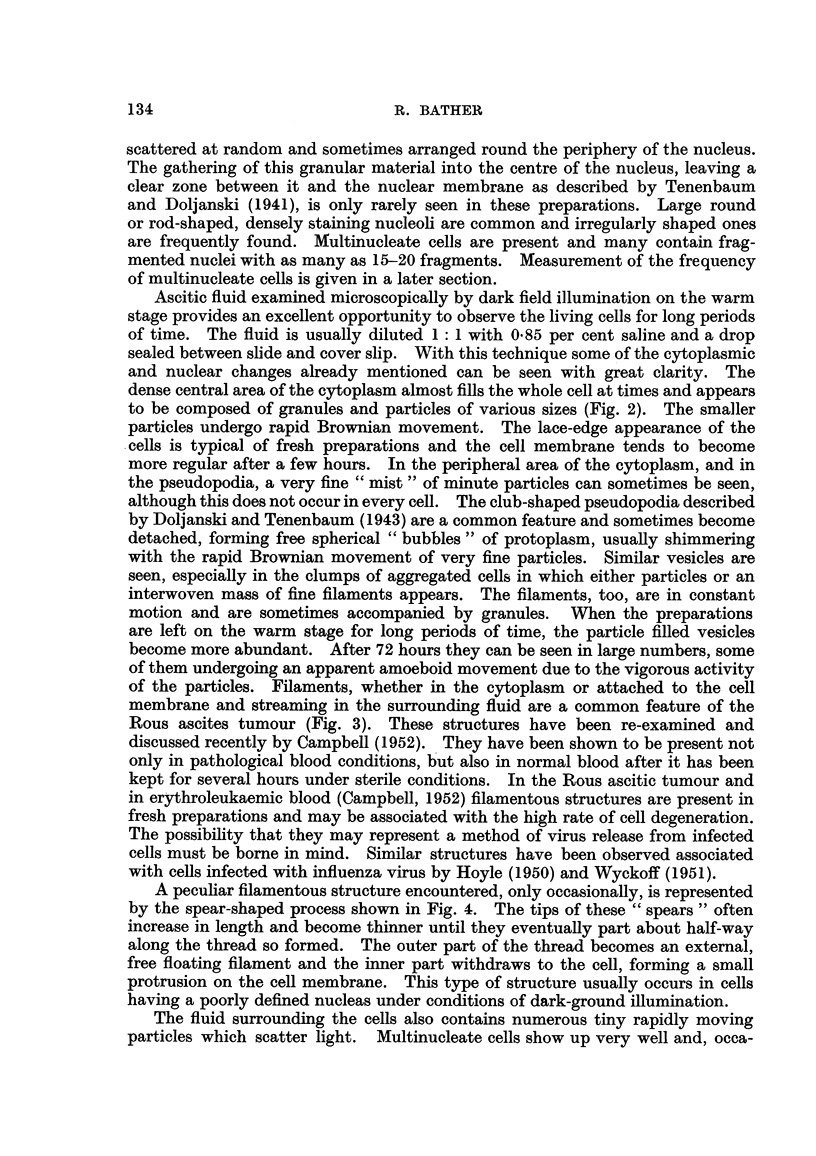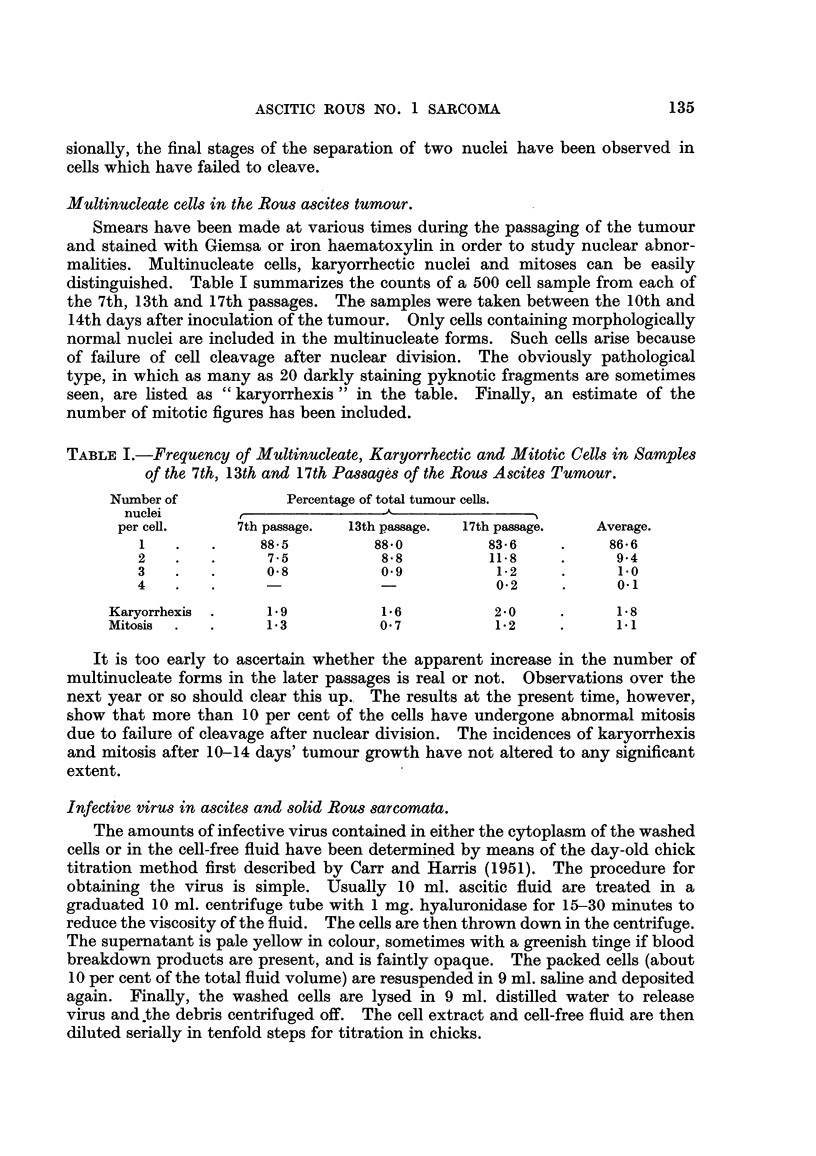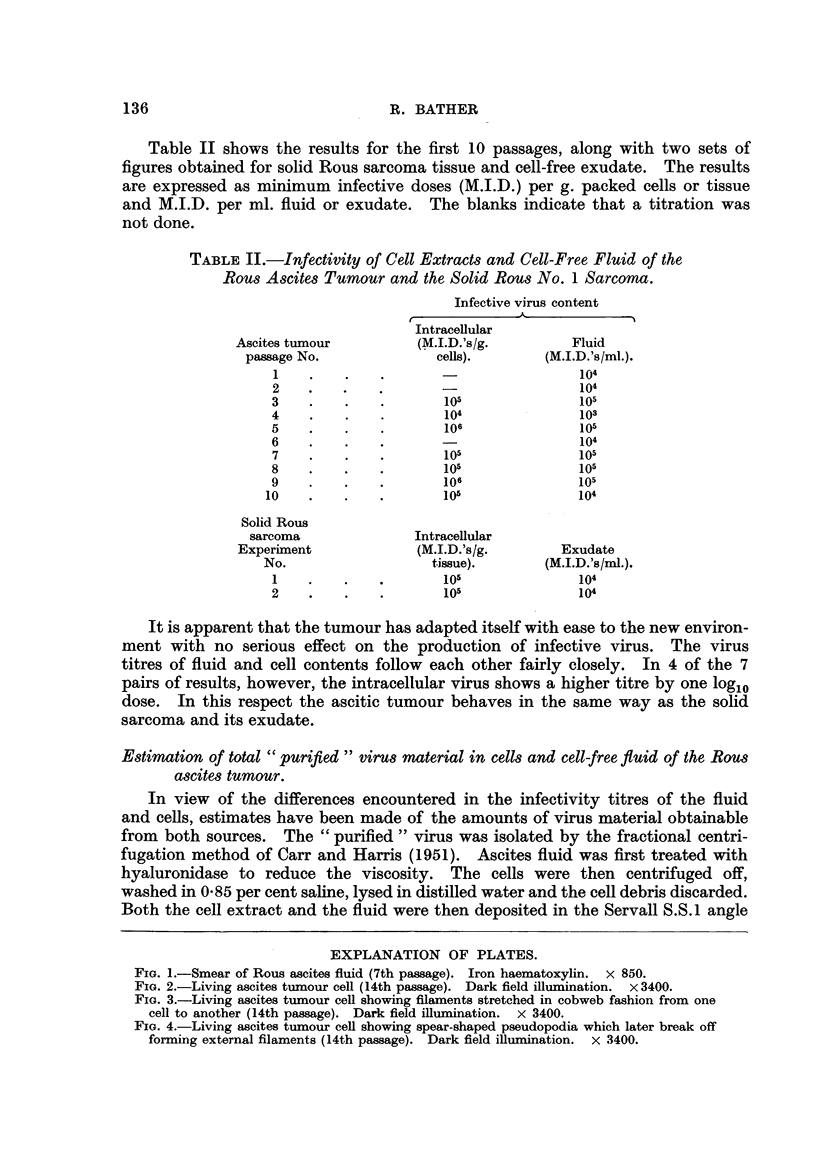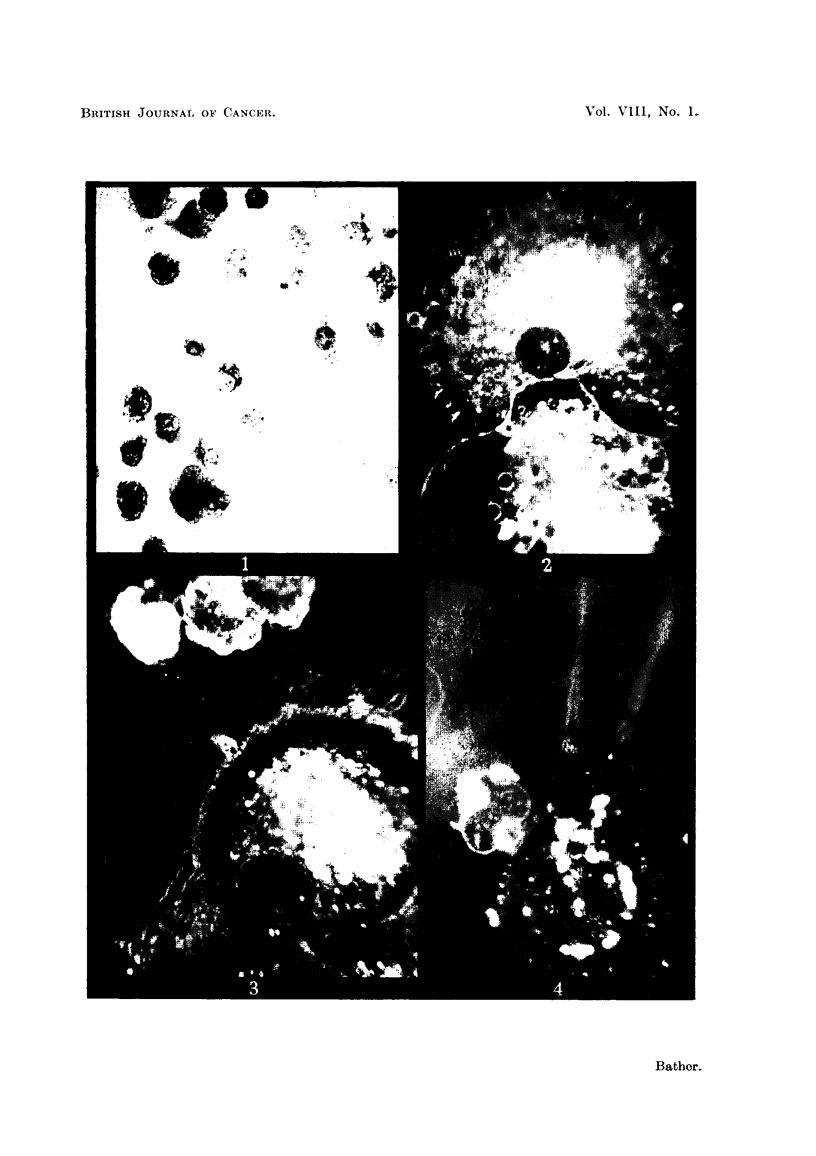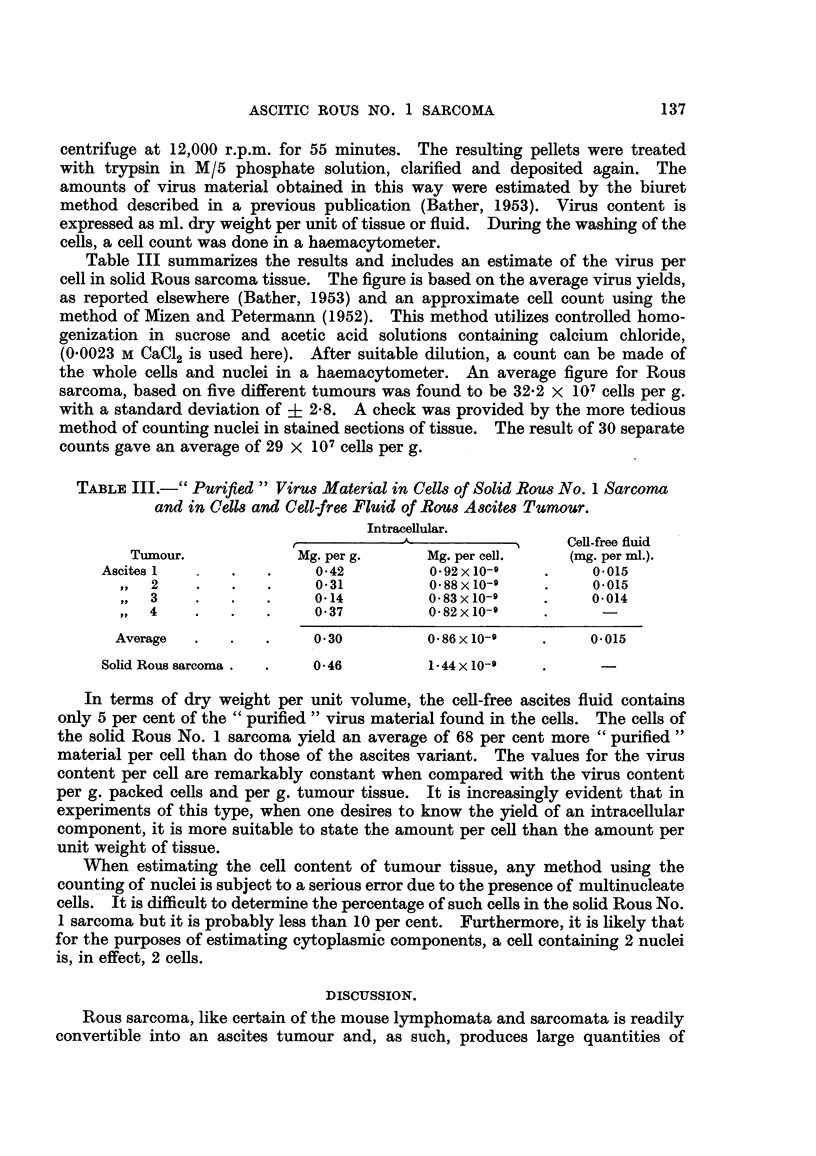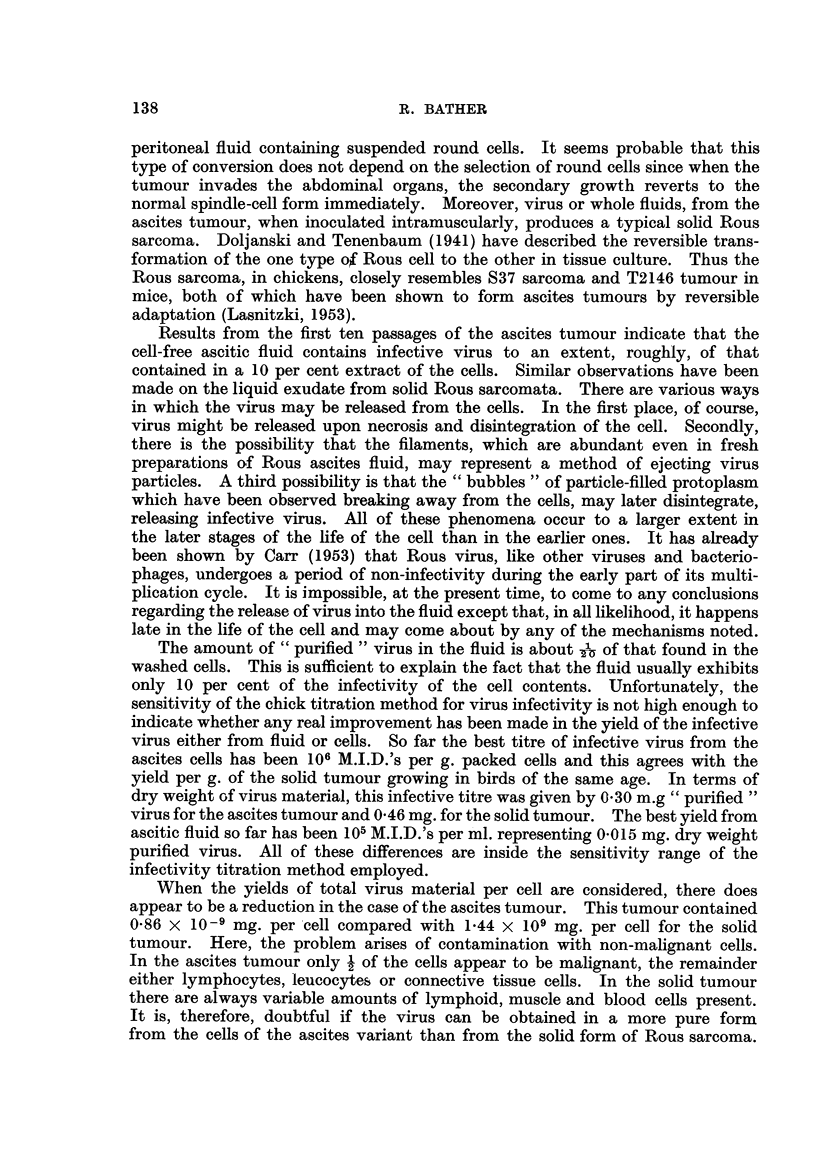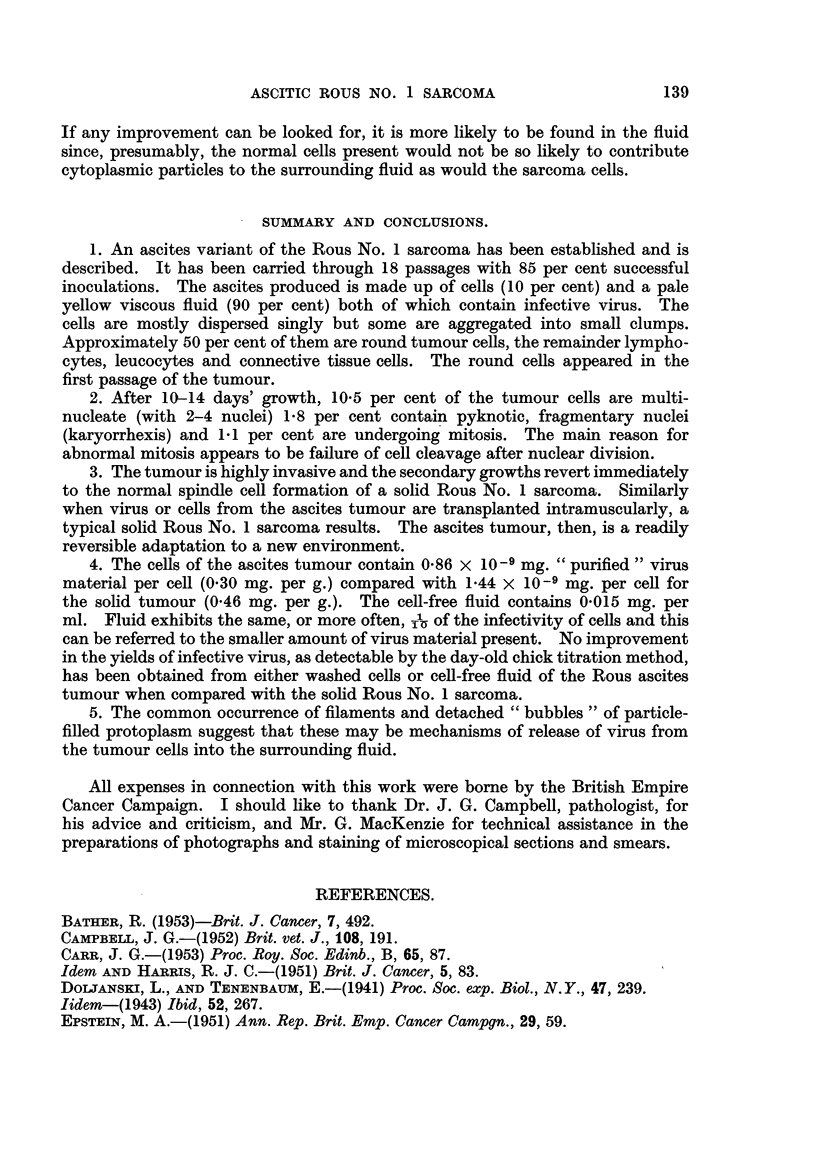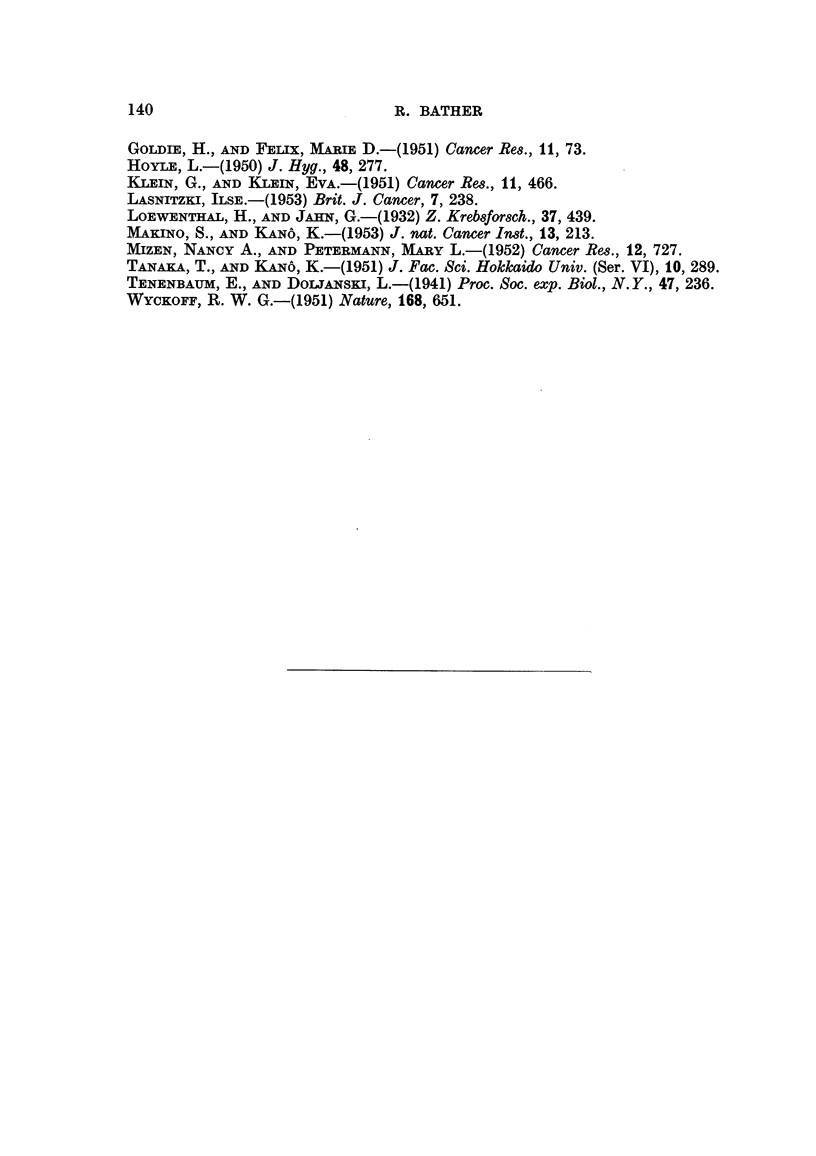# Some Observations on an Ascitic Rous No. 1 Sarcoma

**DOI:** 10.1038/bjc.1954.10

**Published:** 1954-03

**Authors:** R. Bather

## Abstract

**Images:**


### SOME OBSERVATIONS ON AN ASCITIC ROUS NO. 1 SARCOMA.

R. BATHER.

*From the Poultry Research Centre, Edinburgh,* 9.

Received for publication November 18, 1953.

CONSIDERABLE interest has been centred lately on the use of ascites tumours in rats and mice for biological research. Such tumours include, in mice, the Ehrlich and Krebs-2 carcinomata (Loewenthal and Jahn, 1932 ; Klein and Klein, 1951) and S-37 and malignant lymphoma (Goldie and Felix, 1951) and, in rats, the Yoshida sarcoma, the MTK sarcomata I and II (Tanaka and Kanô, 1951) and the Hirosaki sarcoma (Makino and Kanô, 1953). The growth of these tumours is characterized by the formation of large numbers of discrete tumour cells, living in a compatible medium (peritoneal fluid) which lend themselves admirably to various biochemical studies.

During the course of investigations into the nature of the virus of Rous sarcoma, an ascitic variant of the tumour was established early in 1953 in order to see if infective virus could be obtained in a more pure form. The fact that Rous No. 1 sarcoma can be grown intraperitoneally to produce freely dispersed cells and an ascitic fluid containing extracellular virus has already been noted (Epstein, 1951).

### MATERIALS AND METHODS.

### *Animal material.*

All the chicks or chickens used in this work were obtained, by kind permission of Dr. A. W. Greenwood, from his inbred flock of Brown Leghorns maintained at Edinburgh. Other materials and techniques will be described in the experimental sections concerned.

### EXPERIMENTAL.

### *Establishment of the ascites tumour.*

A 10 per cent suspension of cells from a rapidly growing Rous No. 1 sarcoma was prepared by macerating 1·0 g. of the tissue in 0·85 per cent. saline A small, all-glass macerator was used and only firm Rous tissue was selected showing no evidence of necrosis. Using a wide bore needle, 1 ml. of the suspension was inoculated into the abdominal cavity of each of 2 6-week old chickens from a known Rous susceptible strain maintained at this Centre. The needle was introduced into the soft part of the abdomen adjacent to the anterior end of the right pubis. With care, the inoculum can be deposited between the folds of the intestine without penetration of the gizzard or other tissues. The first bird died 17 days after the inoculation and, upon examination, the abdomen was found to contain about 12–15 ml. of mucous ascitic fluid which was removed for inoculation into fresh birds. The second bird was killed 29 days after inoculation and yielded about 30 ml. fluid. Rous tissue was found to have attached itself to the abdominal wall, surface of the gizzard and intestine, and the peritoneum. Several small loosely adherent nodules were to be seen on the surface of the liver.

The tumour has now been carried through 18 passages. So far 84 animals have been inoculated and 71 successful tumours recorded (85 per cent). Most of the negative results can be attributed to failure of the inoculum to enter the abdomen and, usually, subcutaneous tumours grew at the site of entry of the needle.

The rapid growth of the Rous tissue and its invasion of the internal organs has been observed in all subsequent passages. Small nodules of tumour tissue attach themselves to the surface of the organs and then proceed to infiltrate the membrane and musculature, often penetrating to the interior of the organ itself. Of 30 birds from the 1st to the 12th passages autopsied 9–19 days after the inoculation of the tumour, the following tissues showed invasion microscopically and grossly : Gizzard (30), pancreas (27), duodenum (27), spleen (25), ovary (17 out of 20), testis (5 out of 10), liver (12), heart (10), adrenal (10), kidney (7), and lungs (1). In the cases of the ovary and pancreas, invasion has frequently progressed to such an extent that only a few fragments of normal tissue remain, almost the whole organ having been replaced by tumour tissue. Such extensive encroachment has been noted after only 10 days' growth of the tumour.

The ascitic fluid itself, when examined under the microscope is seen to contain mostly individual cells and a few small clumps of cells. There are usually 10–15 ml. pale yellow, viscous fluid present in a 6-week-old bird after 10–12 days' incubation, and, on occasion, as much as 60 ml. have been obtained. The animals generally survive about 12–14 days. The cells account for approximately 10 per cent of the fluid volume. Counts of the cells made from stained smears (Giemsa) at various times during the passaging of the tumour show that of over 2000 cells counted approximately 50 per cent are round tumour cells, and the remainder leukocytes, lymphocytes and connective tissue cells. This proportion has remained unchanged throughout the 18 passages so far maintained. Whenever intensive invasion has occurred, the secondary growth has resumed its normal histology of whorling bundles of spindle cells. On various occasions, virus or whole cells from ascitic fluid have been inoculated into the muscles of birds and, again, there is an immediate return to the normal histological picture of a solid Rous No. 1 sarcoma.

*Cytoplasmic and nuclear characteristics of ascitic Rous sarcoma cells.*

Smears of the ascitic fluid have been made and stained either by the Giemsa, Feulgen or iron haematoxylin method for examination in the microscope. Fig. 1 shows the typical appearance of the fluid using iron haematoxylin stain. The cytoplasmic and nuclear changes in Rous sarcoma cells cultivated *in vitro* have been described (Tenenbaum and Doljanski, 1941 ; Doljanski and Tenenbaum, 1943) and many of the descriptions apply to the appearance of the cells grown intraperitoneally. The most obvious feature is the tremendous range in size of the cells. Most of them are 8–12$\mu$ in diameter when smeared and stained with iron haematoxylin, but some have been observed with a diameter of 22$\mu$. The average length of the chicken red blood cell in the same smears is 11$\mu$. The blue staining granule and thread network in the peripheral cytoplasm (Giemsa stain) and the dense central zone can be seen, as well as the large cytoplasmic vacuoles and club-shaped pseudopodia. The nuclei often show many particulate masses, sometimes scattered at random and sometimes arranged round the periphery of the nucleus. The gathering of this granular material into the centre of the nucleus, leaving a clear zone between it and the nuclear membrane as described by Tenenbaum and Doljanski (1941), is only rarely seen in these preparations. Large round or rod-shaped, densely staining nucleoli are common and irregularly shaped ones are frequently found. Multinucleate cells are present and many contain fragmented nuclei with as many as 15–20 fragments. Measurement of the frequency of multinucleate cells is given in a later section.

Ascitic fluid examined microscopically by dark field illumination on the warm stage provides an excellent opportunity to observe the living cells for long periods of time. The fluid is usually diluted 1 : 1 with 0·85 per cent saline and a drop sealed between slide and cover slip. With this technique some of the cytoplasmic and nuclear changes already mentioned can be seen with great clarity. The dense central area of the cytoplasm almost fills the whole cell at times and appears to be composed of granules and particles of various sizes (Fig. 2). The smaller particles undergo rapid Brownian movement. The lace-edge appearance of the cells is typical of fresh preparations and the cell membrane tends to become more regular after a few hours. In the peripheral area of the cytoplasm, and in the pseudopodia, a very fine " mist " of minute particles can sometimes be seen, although this does not occur in every cell. The club-shaped pseudopodia described by Doljanski and Tenenbaum (1943) are a common feature and sometimes become detached, forming free spherical " bubbles " of protoplasm, usually shimmering with the rapid Brownian movement of very fine particles. Similar vesicles are seen, especially in the clumps of aggregated cells in which either particles or an interwoven mass of fine filaments appears. The filaments, too, are in constant motion and are sometimes accompanied by granules. When the preparations are left on the warm stage for long periods of time, the particle filled vesicles become more abundant. After 72 hours they can be seen in large numbers, some of them undergoing an apparent amoeboid movement due to the vigorous activity of the particles. Filaments, whether in the cytoplasm or attached to the cell membrane and streaming in the surrounding fluid are a common feature of the Rous ascites tumour (Fig. 3). These structures have been re-examined and discussed recently by Campbell (1952). They have been shown to be present not only in pathological blood conditions, but also in normal blood after it has been kept for several hours under sterile conditions. In the Rous ascitic tumour and in erythroleukaemic blood (Campbell, 1952) filamentous structures are present in fresh preparations and may be associated with the high rate of cell degeneration. The possibility that they may represent a method of virus release from infected cells must be borne in mind. Similar structures have been observed associated with cells infected with influenza virus by Hoyle (1950) and Wyckoff (1951).

A peculiar filamentous structure encountered, only occasionally, is represented by the spear-shaped process shown in Fig. 4. The tips of these " spears " often increase in length and become thinner until they eventually part about half-way along the thread so formed. The outer part of the thread becomes an external, free floating filament and the inner part withdraws to the cell, forming a small protrusion on the cell membrane. This type of structure usually occurs in cells having a poorly defined nucleas under conditions of dark-ground illumination.

The fluid surrounding the cells also contains numerous tiny rapidly moving particles which scatter light. Multinucleate cells show up very well and, occasionally, the final stages of the separation of two nuclei have been observed in cells which have failed to cleave.

*Multinucleate cells in the Rous ascites tumour.*

Smears have been made at various times during the passaging of the tumour and stained with Giemsa or iron haematoxylin in order to study nuclear abnormalities. Multinucleate cells, karyorrhectic nuclei and mitoses can be easily distinguished. Table I summarizes the counts of a 500 cell sample from each of the 7th, 13th and 17th passages. The samples were taken between the 10th and 14th days after inoculation of the tumour. Only cells containing morphologically normal nuclei are included in the multinucleate forms. Such cells arise because of failure of cell cleavage after nuclear division. The obviously pathological type, in which as many as 20 darkly staining pyknotic fragments are sometimes seen, are listed as " karyorrhexis " in the table. Finally, an estimate of the number of mitotic figures has been included.

TABLE I.—*Frequency of Multinucleate, Karyorrhectic and Mitotic Cells in Samples of the 7th, 13th and 17th Passages of the Rous Ascites Tumour.*

| Number of nuclei per cell. | Percentage of total tumour cells. | | | |
|---|---|---|---|---|
| | 7th passage. | 13th passage. | 17th passage. | Average. |
| 1 | 88·5 | 88·0 | 83·6 | 86·6 |
| 2 | 7·5 | 8·8 | 11·8 | 9·4 |
| 3 | 0·8 | 0·9 | 1·2 | 1·0 |
| 4 | — | — | 0·2 | 0·1 |
| Karyorrhexis | 1·9 | 1·6 | 2·0 | 1·8 |
| Mitosis | 1·3 | 0·7 | 1·2 | 1·1 |

It is too early to ascertain whether the apparent increase in the number of multinucleate forms in the later passages is real or not. Observations over the next year or so should clear this up. The results at the present time, however, show that more than 10 per cent of the cells have undergone abnormal mitosis due to failure of cleavage after nuclear division. The incidences of karyorrhexis and mitosis after 10–14 days' tumour growth have not altered to any significant extent.

*Infective virus in ascites and solid Rous sarcomata.*

The amounts of infective virus contained in either the cytoplasm of the washed cells or in the cell-free fluid have been determined by means of the day-old chick titration method first described by Carr and Harris (1951). The procedure for obtaining the virus is simple. Usually 10 ml. ascitic fluid are treated in a graduated 10 ml. centrifuge tube with 1 mg. hyaluronidase for 15–30 minutes to reduce the viscosity of the fluid. The cells are then thrown down in the centrifuge. The supernatant is pale yellow in colour, sometimes with a greenish tinge if blood breakdown products are present, and is faintly opaque. The packed cells (about 10 per cent of the total fluid volume) are resuspended in 9 ml. saline and deposited again. Finally, the washed cells are lysed in 9 ml. distilled water to release virus and the debris centrifuged off. The cell extract and cell-free fluid are then diluted serially in tenfold steps for titration in chicks.

Table II shows the results for the first 10 passages, along with two sets of figures obtained for solid Rous sarcoma tissue and cell-free exudate. The results are expressed as minimum infective doses (M.I.D.) per g. packed cells or tissue and M.I.D. per ml. fluid or exudate. The blanks indicate that a titration was not done.

TABLE II.—*Infectivity of Cell Extracts and Cell-Free Fluid of the Rous Ascites Tumour and the Solid Rous No. 1 Sarcoma.*

| | Infective virus content | |
|---|---|---|
| Ascites tumour passage No. | Intracellular (M.I.D.'s/g. cells). | Fluid (M.I.D.'s/ml.). |
| 1 . . . | — | $10^4$ |
| 2 . . . | — | $10^4$ |
| 3 . . . | $10^5$ | $10^5$ |
| 4 . . . | $10^4$ | $10^3$ |
| 5 . . . | $10^6$ | $10^5$ |
| 6 . . . | — | $10^4$ |
| 7 . . . | $10^5$ | $10^5$ |
| 8 . . . | $10^5$ | $10^5$ |
| 9 . . . | $10^6$ | $10^5$ |
| 10 . . . | $10^5$ | $10^4$ |
| Solid Rous sarcoma Experiment No. | Intracellular (M.I.D.'s/g. tissue). | Exudate (M.I.D.'s/ml.). |
| 1 . . . | $10^5$ | $10^4$ |
| 2 . . . | $10^5$ | $10^4$ |

It is apparent that the tumour has adapted itself with ease to the new environment with no serious effect on the production of infective virus. The virus titres of fluid and cell contents follow each other fairly closely. In 4 of the 7 pairs of results, however, the intracellular virus shows a higher titre by one $\log_{10}$ dose. In this respect the ascitic tumour behaves in the same way as the solid sarcoma and its exudate.

*Estimation of total " purified " virus material in cells and cell-free fluid of the Rous ascites tumour.*

In view of the differences encountered in the infectivity titres of the fluid and cells, estimates have been made of the amounts of virus material obtainable from both sources. The " purified " virus was isolated by the fractional centrifugation method of Carr and Harris (1951). Ascites fluid was first treated with hyaluronidase to reduce the viscosity. The cells were then centrifuged off, washed in 0·85 per cent saline, lysed in distilled water and the cell debris discarded. Both the cell extract and the fluid were then deposited in the Servall S.S.1 angle

EXPLANATION OF PLATES.

FIG. 1.—Smear of Rous ascites fluid (7th passage). Iron haematoxylin. × 850.
FIG. 2.—Living ascites tumour cell (14th passage). Dark field illumination. ×3400.
FIG. 3.—Living ascites tumour cell showing filaments stretched in cobweb fashion from one cell to another (14th passage). Dark field illumination. × 3400.
FIG. 4.—Living ascites tumour cell showing spear-shaped pseudopodia which later break off forming external filaments (14th passage). Dark field illumination. × 3400.

1 2 3 4

Bather.

centrifuge at 12,000 r.p.m. for 55 minutes. The resulting pellets were treated with trypsin in M/5 phosphate solution, clarified and deposited again. The amounts of virus material obtained in this way were estimated by the biuret method described in a previous publication (Bather, 1953). Virus content is expressed as ml. dry weight per unit of tissue or fluid. During the washing of the cells, a cell count was done in a haemacytometer.

Table III summarizes the results and includes an estimate of the virus per cell in solid Rous sarcoma tissue. The figure is based on the average virus yields, as reported elsewhere (Bather, 1953) and an approximate cell count using the method of Mizen and Petermann (1952). This method utilizes controlled homogenization in sucrose and acetic acid solutions containing calcium chloride, (0·0023 M $CaCl_2$ is used here). After suitable dilution, a count can be made of the whole cells and nuclei in a haemacytometer. An average figure for Rous sarcoma, based on five different tumours was found to be $32{\cdot}2 \times 10^7$ cells per g. with a standard deviation of $\pm$ 2·8. A check was provided by the more tedious method of counting nuclei in stained sections of tissue. The result of 30 separate counts gave an average of $29 \times 10^7$ cells per g.

TABLE III.—" *Purified* " *Virus Material in Cells of Solid Rous No.* 1 *Sarcoma and in Cells and Cell-free Fluid of Rous Ascites Tumour.*

| Tumour. | Intracellular. | | Cell-free fluid |
|---|---|---|---|
| | Mg. per g. | Mg. per cell. | (mg. per ml.). |
| Ascites 1 | 0·42 | $0{\cdot}92 \times 10^{-9}$ | 0·015 |
| „ 2 | 0·31 | $0{\cdot}88 \times 10^{-9}$ | 0·015 |
| „ 3 | 0·14 | $0{\cdot}83 \times 10^{-9}$ | 0·014 |
| „ 4 | 0·37 | $0{\cdot}82 \times 10^{-9}$ | — |
| Average | 0·30 | $0{\cdot}86 \times 10^{-9}$ | 0·015 |
| Solid Rous sarcoma | 0·46 | $1{\cdot}44 \times 10^{-9}$ | — |

In terms of dry weight per unit volume, the cell-free ascites fluid contains only 5 per cent of the " purified " virus material found in the cells. The cells of the solid Rous No. 1 sarcoma yield an average of 68 per cent more " purified " material per cell than do those of the ascites variant. The values for the virus content per cell are remarkably constant when compared with the virus content per g. packed cells and per g. tumour tissue. It is increasingly evident that in experiments of this type, when one desires to know the yield of an intracellular component, it is more suitable to state the amount per cell than the amount per unit weight of tissue.

When estimating the cell content of tumour tissue, any method using the counting of nuclei is subject to a serious error due to the presence of multinucleate cells. It is difficult to determine the percentage of such cells in the solid Rous No. 1 sarcoma but it is probably less than 10 per cent. Furthermore, it is likely that for the purposes of estimating cytoplasmic components, a cell containing 2 nuclei is, in effect, 2 cells.

### DISCUSSION.

Rous sarcoma, like certain of the mouse lymphomata and sarcomata is readily convertible into an ascites tumour and, as such, produces large quantities of peritoneal fluid containing suspended round cells. It seems probable that this type of conversion does not depend on the selection of round cells since when the tumour invades the abdominal organs, the secondary growth reverts to the normal spindle-cell form immediately. Moreover, virus or whole fluids, from the ascites tumour, when inoculated intramuscularly, produces a typical solid Rous sarcoma. Doljanski and Tenenbaum (1941) have described the reversible transformation of the one type of Rous cell to the other in tissue culture. Thus the Rous sarcoma, in chickens, closely resembles S37 sarcoma and T2146 tumour in mice, both of which have been shown to form ascites tumours by reversible adaptation (Lasnitzki, 1953).

Results from the first ten passages of the ascites tumour indicate that the cell-free ascitic fluid contains infective virus to an extent, roughly, of that contained in a 10 per cent extract of the cells. Similar observations have been made on the liquid exudate from solid Rous sarcomata. There are various ways in which the virus may be released from the cells. In the first place, of course, virus might be released upon necrosis and disintegration of the cell. Secondly, there is the possibility that the filaments, which are abundant even in fresh preparations of Rous ascites fluid, may represent a method of ejecting virus particles. A third possibility is that the " bubbles " of particle-filled protoplasm which have been observed breaking away from the cells, may later disintegrate, releasing infective virus. All of these phenomena occur to a larger extent in the later stages of the life of the cell than in the earlier ones. It has already been shown by Carr (1953) that Rous virus, like other viruses and bacteriophages, undergoes a period of non-infectivity during the early part of its multiplication cycle. It is impossible, at the present time, to come to any conclusions regarding the release of virus into the fluid except that, in all likelihood, it happens late in the life of the cell and may come about by any of the mechanisms noted.

The amount of " purified " virus in the fluid is about $\frac{1}{20}$ of that found in the washed cells. This is sufficient to explain the fact that the fluid usually exhibits only 10 per cent of the infectivity of the cell contents. Unfortunately, the sensitivity of the chick titration method for virus infectivity is not high enough to indicate whether any real improvement has been made in the yield of the infective virus either from fluid or cells. So far the best titre of infective virus from the ascites cells has been $10^6$ M.I.D.'s per g. packed cells and this agrees with the yield per g. of the solid tumour growing in birds of the same age. In terms of dry weight of virus material, this infective titre was given by 0·30 m.g " purified " virus for the ascites tumour and 0·46 mg. for the solid tumour. The best yield from ascitic fluid so far has been $10^5$ M.I.D.'s per ml. representing 0·015 mg. dry weight purified virus. All of these differences are inside the sensitivity range of the infectivity titration method employed.

When the yields of total virus material per cell are considered, there does appear to be a reduction in the case of the ascites tumour. This tumour contained $0{\cdot}86 \times 10^{-9}$ mg. per cell compared with $1{\cdot}44 \times 10^{9}$ mg. per cell for the solid tumour. Here, the problem arises of contamination with non-malignant cells. In the ascites tumour only $\frac{1}{2}$ of the cells appear to be malignant, the remainder either lymphocytes, leucocytes or connective tissue cells. In the solid tumour there are always variable amounts of lymphoid, muscle and blood cells present. It is, therefore, doubtful if the virus can be obtained in a more pure form from the cells of the ascites variant than from the solid form of Rous sarcoma.

If any improvement can be looked for, it is more likely to be found in the fluid since, presumably, the normal cells present would not be so likely to contribute cytoplasmic particles to the surrounding fluid as would the sarcoma cells.

### SUMMARY AND CONCLUSIONS.

1. An ascites variant of the Rous No. 1 sarcoma has been established and is described. It has been carried through 18 passages with 85 per cent successful inoculations. The ascites produced is made up of cells (10 per cent) and a pale yellow viscous fluid (90 per cent) both of which contain infective virus. The cells are mostly dispersed singly but some are aggregated into small clumps. Approximately 50 per cent of them are round tumour cells, the remainder lymphocytes, leucocytes and connective tissue cells. The round cells appeared in the first passage of the tumour.

2. After 10–14 days' growth, 10·5 per cent of the tumour cells are multinucleate (with 2–4 nuclei) 1·8 per cent contain pyknotic, fragmentary nuclei (karyorrhexis) and 1·1 per cent are undergoing mitosis. The main reason for abnormal mitosis appears to be failure of cell cleavage after nuclear division.

3. The tumour is highly invasive and the secondary growths revert immediately to the normal spindle cell formation of a solid Rous No. 1 sarcoma. Similarly when virus or cells from the ascites tumour are transplanted intramuscularly, a typical solid Rous No. 1 sarcoma results. The ascites tumour, then, is a readily reversible adaptation to a new environment.

4. The cells of the ascites tumour contain $0{\cdot}86 \times 10^{-9}$ mg. " purified " virus material per cell (0·30 mg. per g.) compared with $1{\cdot}44 \times 10^{-9}$ mg. per cell for the solid tumour (0·46 mg. per g.). The cell-free fluid contains 0·015 mg. per ml. Fluid exhibits the same, or more often, $\frac{1}{10}$ of the infectivity of cells and this can be referred to the smaller amount of virus material present. No improvement in the yields of infective virus, as detectable by the day-old chick titration method, has been obtained from either washed cells or cell-free fluid of the Rous ascites tumour when compared with the solid Rous No. 1 sarcoma.

5. The common occurrence of filaments and detached " bubbles " of particle-filled protoplasm suggest that these may be mechanisms of release of virus from the tumour cells into the surrounding fluid.

All expenses in connection with this work were borne by the British Empire Cancer Campaign. I should like to thank Dr. J. G. Campbell, pathologist, for his advice and criticism, and Mr. G. MacKenzie for technical assistance in the preparations of photographs and staining of microscopical sections and smears.

### REFERENCES.

BATHER, R. (1953)—*Brit. J. Cancer*, **7**, 492.
CAMPBELL, J. G.—(1952) *Brit. vet. J.*, **108**, 191.
CARR, J. G.—(1953) *Proc. Roy. Soc. Edinb.*, B, **65**, 87.
*Idem* AND HARRIS, R. J. C.—(1951) *Brit. J. Cancer*, **5**, 83.
DOLJANSKI, L., AND TENENBAUM, E.—(1941) *Proc. Soc. exp. Biol., N.Y.*, **47**, 239.
*Iidem*—(1943) *Ibid*, **52**, 267.
EPSTEIN, M. A.—(1951) *Ann. Rep. Brit. Emp. Cancer Campgn.*, **29**, 59.

GOLDIE, H., AND FELIX, MARIE D.—(1951) *Cancer Res.*, **11**, 73.
HOYLE, L.—(1950) *J. Hyg.*, **48**, 277.
KLEIN, G., AND KLEIN, EVA.—(1951) *Cancer Res.*, **11**, 466.
LASNITZKI, ILSE.—(1953) *Brit. J. Cancer*, **7**, 238.
LOEWENTHAL, H., AND JAHN, G.—(1932) *Z. Krebsforsch.*, **37**, 439.
MAKINO, S., AND KANÔ, K.—(1953) *J. nat. Cancer Inst.*, **13**, 213.
MIZEN, NANCY A., AND PETERMANN, MARY L.—(1952) *Cancer Res.*, **12**, 727.
TANAKA, T., AND KANÔ, K.—(1951) *J. Fac. Sci. Hokkaido Univ.* (Ser. VI), **10**, 289.
TENENBAUM, E., AND DOLJANSKI, L.—(1941) *Proc. Soc. exp. Biol., N.Y.*, **47**, 236.
WYCKOFF, R. W. G.—(1951) *Nature*, **168**, 651.